# Development and Characterization of High‐Nutrient Biscuits for Disaster Situations Targeting At‐Risk Groups

**DOI:** 10.1155/ijfo/8042259

**Published:** 2025-12-11

**Authors:** Tuğçe Boğa, Kübra Topaloğlu Günan, Seda Çakmak Kavsara, Didem Berber, Özlem Aktürk Gümüşay

**Affiliations:** ^1^ Department of Gastronomy and Culinary Arts, Faculty of Fine Arts, Maltepe University, Istanbul, Türkiye, maltepe.edu.tr

**Keywords:** disaster, emergency, gluten-free biscuit, risk groups

## Abstract

In emergency and disaster situations, access to nutritionally adequate, shelf‐stable, and acceptable food products becomes a critical public health priority. This study is aimed at developing four biscuit formulations designed to meet the specific dietary needs of vulnerable population groups, including the general population, elderly individuals, children, and those requiring a gluten‐free formulation. The biscuits were evaluated for their physical (color and texture), nutritional (protein, fat, ash, carbohydrate, and dietary fiber), and in vitro bioavailability of phenolic content, as well as their sensory acceptability. The biscuit for elderly consumption (BEC) demonstrated the highest ash, fat, protein, and dietary fiber levels, indicating a nutritionally enriched profile suitable for aging populations. Meanwhile, the biscuit for gluten‐free formulation (BGF) contained the highest carbohydrate content but exhibited lower levels of key nutrients, reflecting some nutritional limitations. Sensory analyses confirmed acceptable palatability across all formulations, with texture and flavor identified as key drivers of preference. These results highlight the importance of ingredient selection and functional formulation in the development of emergency foods tailored to the diverse nutritional requirements of at‐risk populations.

## 1. Introduction

Natural disasters often disrupt food systems, making it difficult to establish standardized approaches for emergency nutrition management [1, 2]. Disaster‐affected populations are recommended to receive an energy distribution of approximately 55%–60% of total energy from carbohydrates, 10%–12% from protein, and 25%–30% from fat [3]. The provision of micronutrients should ensure adequate intake of iron (10–18 mg/day), calcium (500–1000 mg/day), vitamin A (500–600 *μ*g RE/day), and vitamin C (45–90 mg/day), varying by population group and physiological requirements [3–8].

The first 72 h following a disaster constitute the short‐term nutrition phase, during which emergency food assistance is essential [5]. However, these provisions frequently overlook the specific needs of vulnerable groups such as young children, pregnant women, older adults, and individuals with chronic diseases or food intolerances, resulting in suboptimal nutritional support [1]. Existing emergency foods largely prioritize energy density and storage stability, underscoring the need for specialized products that are both safe and nutritionally adequate for at‐risk populations, including older adults who require higher protein and micronutrient intakes and individuals requiring gluten‐free alternatives [9, 10]. Recent guidance consolidates practical principles for emergency nutrition, highlighting energy and protein adequacy, micronutrient provision, shelf stability, and logistical feasibility in household food kits [7]. In parallel, the disaster–food literature often treats packaging primarily from a shelf‐life standpoint, focusing on barrier properties and time‐dependent stability [11]. At the product level, biscuit‐type emergency foods have been explored as compact, ready‐to‐eat carriers; for example, Anandito et al. optimized proso millet biscuits enriched with snakehead fish–tempeh “koya,” reporting acceptable sensory attributes together with favorable proximate composition and energy density [6].

Biscuits are widely consumed bakery products due to their sensory appeal, convenience, long shelf life, affordability, and broad acceptance across age groups [12]. Research indicates that their nutritional, physicochemical, and sensory properties can be improved by enriching formulations with functional components [13]. Recent studies have focused on enhancing the nutritional profile of biscuits through the incorporation of alternative ingredients. Various flours (e.g., chickpea, rice, oat, buckwheat, flaxseed, mulberry, moringa, and psyllium) and hydrocolloids (e.g., xanthan gum and chia gel) have been used to increase micronutrients and provide gluten‐free options [14–18]. To improve dietary fiber content, ingredients like fruit and vegetable peels, seed flours, legume‐based flours, and coffee bean membranes have been utilized [19–22]. Given the health risks associated with high sugar intake, particularly among children and older adults [13], natural sweeteners such as stevia have been investigated. Stevia offers a nonglycemic alternative suitable for individuals with diabetes [23, 24].

This study addresses a key gap by developing four ready‐to‐eat, energy‐dense biscuit formulations tailored to the general population and vulnerable groups (children, older adults, and individuals requiring gluten‐free options) and by linking the formulation strategy to nutritional quality, in vitro bioaccessibility of phenolics, sensory performance, and microbiological status. The formulations were engineered to enhance physical, textural, and sensory properties, with processing conditions selected to improve structure, nutritional quality, and retention of functional ingredients. The overarching aim is to provide high‐nutrient, population‐responsive products for disaster response and early recovery through targeted formulation and processing choices that increase dietary fiber, preserve functional components, and optimize physical and textural characteristics for broad acceptability.

## 2. Materials and Methods

### 2.1. Materials

Ferrous sulfate used in the formulation was obtained from Beyan Lab. Ürünleri San. and Tic. Ltd. Şti. (Istanbul, Türkiye). Other ingredients, including whole wheat flour (WWF) (Sinangil, Tekirdağ, Türkiye), wheat germ (WG) (OG Store, Sakarya, Türkiye), mulberry powder (MP) (Talya Foods, Antalya, Türkiye), pumpkin seed flour (PSF) (Naturdan, İzmir, Türkiye), rice flour (RF) (Dr. Oetker, Izmir, Türkiye), oat flour (OF) (Talya Foods, Antalya, Türkiye), extra virgin olive oil (Tariş, İzmir, Türkiye), powdered sugar (Dr. Oetker, Izmir, Türkiye), stevia leaf powder (Takita, İzmir, Türkiye), date juice (OG Store, Sakarya, Türkiye), sodium bicarbonate (Akbel Kimya, Bursa, Türkiye), and salt (Billur Tuz, İzmir, Türkiye), were purchased from local markets in Istanbul.

### 2.2. Preparation of Biscuit Samples

The biscuit formulations were developed by modifying the method of Büyük and Dulger Altiner [25] based on a comprehensive literature review. As shown in Table 1, four different biscuit formulations were prepared: a biscuit for general consumption (BGC), a biscuit for elderly consumption (BEC), a biscuit for children′s consumption (BCC), and a biscuit for gluten‐free formulation (BGF) developed to meet the needs of individuals with gluten intolerance, regardless of age group. The formulations were designed considering the distinct nutritional requirements of different population groups. The estimated daily energy requirements were approximately 2100 kcal for general adults, 1800 kcal for elderly individuals, and 1600 kcal for children, based on WHO/FAO and Sphere Handbook recommendations [1, 2, 4]. The recommended energy distribution was approximately 55%–60% of total energy from carbohydrates, 10%–12% from protein, and 25%–30% from fat for adults; 50%–55% carbohydrates, 12%–15% protein, and 30%–35% fat for the elderly; and 55%–60% carbohydrates, 12%–14% protein, and 25%–30% fat for children [1, 2]. Furthermore, micronutrient requirements such as iron (10–18 mg/day), calcium (500–1000 mg/day), vitamin A (500–600 *μ*g RE/day), and vitamin C (45–90 mg/day) were considered according to age‐related physiological needs [2]. Accordingly, the proportions of macronutrients and functional ingredients were adjusted to align with these dietary requirements, ensuring balanced energy density and nutrient composition for each formulation. The ingredients were selected based on their nutritional quality and functional contribution to the final product. WWF and WG were used as sources of dietary fiber, essential fatty acids, and micronutrients [26]. Mulberry flour (MF) and PSF were included to enhance antioxidant content, protein quality, and mineral composition, while RF and OF were used to improve texture and provide gluten‐free options with complex carbohydrates [27, 28]. Ferrous sulfate was incorporated as a fortifying agent to improve the iron content of the biscuits, addressing potential micronutrient deficiencies [29]. Based on WHO recommendations of 30–60 mg/day elemental iron for children ≤ 12 years, ferrous sulfate heptahydrate (FeSO_4_·7H_2_O) was used at 0.30 g per 100 g of product, providing approximately 30 mg elemental iron per 50 g serving [30].

**Table 1:** Ingredients used for the biscuit doughs.

**Ingredients (g)**	**BGC**	**BEC**	**BCC**	**BGF**
WWF	80	70	80	0
WG	20	20	20	0
MF	0	0	0	20
PSF	0	10	0	0
RF	0	0	0	40
OF	0	0	0	40
Extra virgin olive oil	40	40	40	40
Powdered sugar	40	20	0	40
Stevia powder	0	0.1	0	0
Date syrup	0	0	30	0
Ferrous sulfate	0	0	0.3	0
Sodium bicarbonate	1.0	1.0	1.0	1.0
Salt	1.0	1.0	1.0	1.0
Water	25	25	25	25

Abbreviations: BCC, biscuit recipe for children′s consumption; BEC, biscuit recipe for elderly consumption; BGC, biscuit recipe for general consumption; BGF, biscuit for gluten‐free formulation; MF, mulberry flour; OF, oat flour; PSF, pumpkin seed flour; RF, rice flour; WG, wheat germ; WWF, whole wheat flour.

For dough preparation, the liquid ingredients were mixed using a stand mixer (Kitchen–Aid Artisan, United States) with a paddle attachment at medium speed (135 rpm) for 2 min. The dry ingredients were then added and mixed at low speed (95 rpm) for an additional 2 min. In the BCC formulation, ferrous sulfate was dissolved in the recipe′s water content before incorporation. The dough was then rolled to a thickness of 0.3 cm between two sheets of baking paper and cut into 6 cm diameter circles using a biscuit cutter. The shaped dough was transferred to baking trays and refrigerated at +4°C for 30 min. Baking was performed in a preheated industrial convection oven (Inoksan INO–FKE010, Türkiye) at 170^°^C ± 2^°^C for 12 min. After baking, the biscuits were rested on the tray for 5 min and then transferred to a cooling rack at room temperature.

### 2.3. Physical and Mechanical Properties of Biscuit Samples

The diameter and thickness of the biscuits were measured 24 h after baking, using a digital caliper according to AACCI Method No. 10.54. The spread ratio was calculated as the ratio of diameter to thickness [31]. Baking loss (%) was determined according to Equation (1), following the procedure described by Bozdogan et al. [32].

(1) Baking loss %=dough weight−baked biscuit weightdough weight×100.

This parameter reflects the mass loss during baking, mainly due to water evaporation and volatilization of certain compounds.

Color analysis was performed using a Minolta Chroma Meter CR‐400 (Minolta, Osaka, Japan) based on the CIE Lab ^∗^ color system. In this system, *L* ^∗^ indicates lightness, *a* ^∗^ indicates redness/greenness, and *b* ^∗^ indicates yellowness/blueness.

Texture analysis was conducted 24 h after baking using the texture profile analysis (TPA) method. A TA‐XT Plus texture analyzer (Stable Micro Systems, United Kingdom) equipped with a three‐point bend ring probe (HDP/3PB) and a heavy‐duty platform (HDP/90) was used to determine the breaking force (*F*, kg) of biscuit samples. The test parameters were load cell = 5 kg, pretest speed = 1.0 mm/s, test speed = 3.0 mm/s, posttest speed = 10.0 mm/s, distance = 5 mm, and trigger force = 50 g. Hardness (g) and fracturability values were measured as textural properties.

### 2.4. Chemical Properties of Biscuit Samples

Moisture content was determined by drying 5 g of ground biscuit samples in preweighed aluminum moisture dishes at 105^°^C ± 2^°^C in a hot air oven (Memmert UN110, Memmert GmbH + Co. KG, Schwabach, Germany) until constant weight. Moisture content was calculated based on the weight loss during drying. Ash content was determined by incinerating the samples at 550°C in a muffle furnace (Nabertherm LV 9/11, Germany), following NMKL No. 173 [33]. Protein content was measured using a Dumas Nitrogen Analyzer (Velp NDA 701, Italy) in accordance with AOAC [34]. Total dietary fiber content was determined using the enzymatic–gravimetric method according to AOAC [35]. Total fat content was analyzed following NMKL No. 160 [36], which involves acid hydrolysis with HCl, fat extraction with petroleum ether, and solvent evaporation using an automatic fat analyzer (Velp SER 148/6, Italy). Carbohydrate and energy values of the biscuit samples were calculated based on the FAO [37] guidelines. All analyses were conducted in triplicate.

### 2.5. In Vitro Simulated Gastric and Intestinal Digestion Study

In vitro digestion of the biscuits′ polyphenol content and DPPH scavenging activity was determined by simulation of gastric and intestinal fluid, separately. The US Pharmacopeia [38] was followed in the preparation of the simulated intestinal fluid (SIF) and simulated gastric fluid (SGF). The preparation of SGF and SIF with enzymes and the subsequent treatment of the biscuit samples with these fluids were carried out according to the method of Saikia et al. [39]. To prepare the SGF solution, 3.2 g of porcine stomach mucosa pepsin (powder, ≥ 250 units/mg solid) was combined with 2.0 g of sodium chloride. Then, 7 mL of 37% hydrochloric acid was added to the mixture, and the volume was diluted to 1000 mL with distilled water to maintain a pH of 1.2. In a 10‐mL test tube, 1.4 mL of SGF was added to 0.1 g of the biscuit sample, and the mixture was incubated for 120 min at 80 rpm at 37°C to stimulate the digestion in the gastric fluid. After cooling at room temperature, the solution was filtered, and a 0.2 mol/L sodium hydroxide solution was added to neutralize it. The SIF solution was prepared by dissolving 6.8 g of monobasic potassium phosphate in 250 mL of water. Pancreatin from porcine pancreas (10 g) (8 × USP specifications) and 0.2 N sodium hydroxide (77 mL) were added to this solution. After that, the mixture′s volume was adjusted to 1000 mL, and the pH was kept at 6.8. A 10‐mL test tube containing 2.4 mL of SIF and 0.1 g of biscuit sample was incubated at 36.6°C for 120 min without shaking in order to simulate the digestion in the intestinal fluid. After cooling to ambient temperature, the solution was filtered, and 2 mL of filtrate was mixed with 100 *μ*L of 3 M hydrochloric acid to lower the pH to 1.2 and restrict enzyme activity. The solution was neutralized (pH 7.0) using 900 *μ*L of 0.2 N sodium hydroxide after 15 min. Finally, the apparent phenolic content of both SGF and SIF samples was determined using the Folin–Ciocalteu method [40]. A spectrophotometer (Shimadzu UV‐1240, Tokyo, Japan) was used for measuring absorbance at 765 nm. Gallic acid at different concentrations was used for the calibration curve (*r* ^2^ = 0.9986). Results were expressed as milligram gallic acid equivalent (GAE) per kilogram of sample. Similarly, the DPPH scavenging activity of SGF and SIF samples was performed using the method of Brand‐Williams et al. [41] with some modifications. After adding 3 mL of DPPH solution (0.051 mmol/L) and 100 *μ*L of extract to a test tube, the mixtures were incubated at room temperature for 30 min. Absorbance values were measured with a spectrophotometer at 517 nm. A standard curve was generated using Trolox solutions at different concentrations (*r* ^2^ = 0.9983). Results were expressed as milligram Trolox equivalent per kilogram of sample. Furthermore, the biscuit sample extract was prepared in pure water for 2 h at 37°C without digestion enzymes, and then, the total phenolic content and DPPH scavenging activity of biscuit samples before digestion were determined.

### 2.6. Sensory Analysis of Biscuit Samples

The sensory evaluation form was developed by modifying the protocols used in the studies of Büyük and Dulger Altiner [25] and Yildiz and Gocmen [42], with adjustments tailored to the scope of this research. Sensory analysis was conducted 2 h postbaking by a semitrained panel of 24 individuals (13 females and 11 males, aged 18–47), consisting of students and academicians from the Department of Gastronomy and Culinary Arts. Each biscuit was quartered, randomly coded with three‐digit numbers, and presented in randomized order on white plastic plates. Samples were evaluated based on seven sensory attributes: color, aroma, taste, crispness, softness, stickiness, and overall acceptability, using a nine‐point hedonic scale (1: *extremely poor*; 9: *excellent*). Samples receiving an average score of 5 or higher were considered acceptable for consumption.

### 2.7. Microbiological Analysis

A microbiologic load analysis was conducted, which included counting the total aerobic mesophilic bacteria, yeast, and mold. Each sample (10 g) was taken aseptically and placed in a sterile 90 mL peptone saline solution. A series of threefold dilutions was performed using the sample homogenates for the inoculation of the experimental setup. The total number of mesophilic aerobic bacterial colonies was enumerated on plate count agar (PCA) following incubation at 30^°^C ± 2^°^C for 48 h, while the fungal colonies were counted on potato dextrose agar (PDA) at 25^°^C ± 2^°^C for 5 days. The experiments were conducted on Days 0, 15, and 30.

### 2.8. Statistical Analysis

Statistical analyses were performed using IBM SPSS Statistics software (Version 21.0; IBM Corp., Armonk, New York, United States). All measurements were conducted in triplicate (*n* = 3), and the results were expressed as mean ± standard deviation. Prior to one‐way ANOVA, the data were tested for normality and homogeneity of variances using the Shapiro–Wilk and Levene′s tests, respectively. When significant differences were detected (*p* < 0.05), Tukey′s post hoc test was applied for multiple comparisons. Pearson′s correlation coefficients were calculated to determine the relationships among physicochemical, textural, sensory, and bioactive parameters. Correlation matrices were used to visualize strong positive or negative associations. For pattern recognition and sample similarity, hierarchical cluster analysis (HCA) was performed using *z*‐score standardized data and the agglomerative hierarchical clustering algorithm with average linkage (between‐groups) and Euclidean distance as the proximity measure. The resulting dendrogram was used to interpret the multivariate relationships among the biscuit formulations. HCA was selected for its capacity to visualize similarity patterns and group relationships among formulations without requiring predefined cluster numbers, thereby enabling an objective, data‐driven interpretation of their multivariate relationships.

## 3. Results and Discussion

### 3.1. Physical and Mechanical Properties of Biscuit Samples

**Figure 1:**
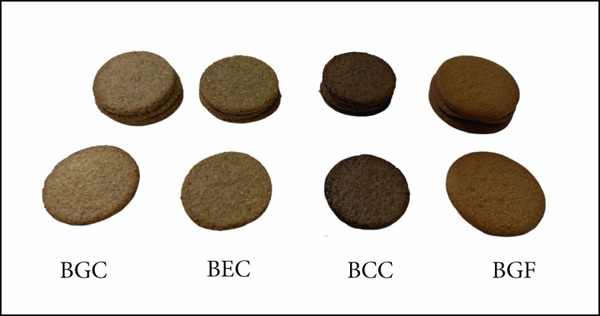
Visual appearance of biscuit samples formulated with different flours.

Biscuit diameter and thickness showed significant variation among formulations (*p* < 0.05) and are presented in Table 2 and Figure 1. The largest diameter was observed in the BGF sample (62.41 mm), while the smallest diameter belonged to BCC (54.20 mm). This suggests that gluten‐free formulations may allow greater dough expansion, possibly due to the high starch content of RF and OF [43]. In terms of thickness, BCC exhibited the greatest value (5.84 mm), which could be associated with its lower spreadability, while BEC had the thinnest profile (4.98 mm). These differences significantly influenced the spread ratio (*D*/*T*), where BCC again showed the lowest value (9.30), indicating lower extensibility and spread, likely due to the presence of ferrous sulfate and higher protein/flour density. The other formulations (BGC, BEC, and BGF) had higher spread ratios than BCC, and there were no statistically significant differences among them (*p* > 0.05).

**Table 2:** Physicochemical characteristics of biscuit samples.

**Tests**		**BGC**	**BEC**	**BCC**	**BGF**
Texture analysis	Hardness (g)	2329.29 ± 345.83^a^	1707.63 ± 217.53^b^	1240.11 ± 146.17^c^	1717.93 ± 179.45^b^
	Fracturability	0.62 ± 0.12^b^	0.65 ± 0.10^b^	0.94 ± 0.13^a^	0.55 ± 0.14^b^

Physical properties	Diameter (mm)	60.92 ± 1.01^ab^	59.07 ± 1.88^b^	54.20 ± 2.00^c^	62.41 ± 0.64^a^
	Thickness (mm)	5.17 ± 0.23^bc^	4.98 ± 0.25^c^	5.84 ± 0.41^a^	5.62 ± 0.23^ab^
	Spread ratio (*D*/*T*)	11.81 ± 0.55^a^	11.87 ± 0.58^a^	9.30 ± 0.51^b^	11.13 ± 0.47^a^
	Baking loss (%)	13.90 ± 2.64^a^	16.33 ± 3.49^a^	14.04 ± 5.02^a^	13.57 ± 2.80^a^

Color analysis	*L* ^∗^	58.49 ± 0.73^a^	56.40 ± 0.52^b^	49.94 ± 1.39^c^	49.45 ± 0.88^c^
	*a* ^∗^	5.34 ± 0.31^c^	5.12 ± 0.26^c^	6.71 ± 0.78^b^	11.29 ± 0.23^a^
	*b* ^∗^	24.50 ± 0.56^ab^	23.53 ± 0.66^b^	21.72 ± 1.30^c^	25.87 ± 0.59^a^

*Note:* Data are the mean ± standard deviation. Different letters in the same line are significantly different (*p* < 0.05).

Abbreviations: BCC, biscuit recipe for children′s consumption; BEC, biscuit recipe for elderly consumption; BGC, biscuit recipe for general consumption; BGF, biscuit for gluten‐free formulation.

The baking loss values of the biscuit samples are presented in Table 2. The results showed that BGC, BEC, BCC, and BGF had baking loss percentages of 13.90%, 16.33%, 14.04%, and 13.57%, respectively. Although no statistically significant difference was observed among the formulations (*p* > 0.05), BEC exhibited slightly higher baking loss, possibly due to its reduced sugar content and higher proportion of PSF, which may have limited moisture retention during baking. Conversely, BGF showed the lowest baking loss, which could be associated with its gluten‐free composition and relatively higher starch content that retains more water during gelatinization. These results are in agreement with previous findings suggesting that fiber‐ and protein‐enriched formulations tend to show reduced mass loss during baking [44, 45].

The textural, physical, and color characteristics of the biscuit samples are provided in Table 2. Among the samples, BGC had the highest hardness value (2329.29 g), while BCC was the softest (1240.11 g). No significant difference was observed between the hardness values of BEC and BGF (*p* > 0.05). Fracturability was significantly higher in BCC (0.94), suggesting increased brittleness. These changes in structural characteristics and hydration dynamics are influenced by the form of iron used in fortification, with recent studies exploring advanced technologies such as iron–casein complexes and encapsulation to mitigate potential interactions with the food matrix [46]. Other samples exhibited similar, lower fracturability values, indicating more cohesive textures.

Color values (*L* ^∗^, *a* ^∗^, and *b* ^∗^) were markedly influenced by the formulation. The BGC biscuit had the highest *L* ^∗^ value (58.49), indicating the lightest color. Conversely, BGF and BCC were significantly darker, potentially due to the presence of MP (high in anthocyanins) and ferrous sulfate, respectively [47]. In *a* ^∗^ (redness) values, BGF showed the highest redness (11.29), consistent with the use of pigmented MP. BCC also had elevated redness compared to BGC and BEC. For *b* ^∗^ (yellowness), BGF again had the highest value (25.87), supporting its more intense yellow hue—possibly due to OF and RF coloration. BCC had the lowest *b* value (21.72), in line with its darker appearance and lower consumer preference in color attributes (as noted in sensory results).

### 3.2. Chemical Properties of Biscuit Samples

Ash content, which serves as an indicator of the total mineral content in food products, varied significantly across the biscuit formulations. The highest ash level was recorded in the BEC sample (1.92/100 g), while the lowest was found in BGF (1.19/100 g) (Table 3). The elevated ash content in BEC can be attributed to the incorporation of nutrient‐rich flours such as PSF and potentially other composite cereal– or legume‐based ingredients, which are known to contain substantial amounts of macro‐ and microminerals, including potassium, magnesium, phosphorus, and iron [48]. These ingredients are frequently utilized in functional food formulations aimed at older adults, not only to enhance nutritional density but also to support physiological needs such as bone health and cardiovascular function. In contrast, the lower ash content in the gluten‐free biscuit (BGF) likely reflects the use of refined or starch‐based gluten‐free flours, which tend to have diminished mineral content unless specifically enriched or fortified [49]. However, it should be noted that the mineral content in this study was not directly analyzed. Therefore, the observed ash content reflects an indirect indication of total mineral presence rather than specific mineral quantification.

**Table 3:** Nutritional composition of biscuit samples (g/100 g biscuit).

	**Ash**	**Moisture**	**Fat**	**Protein**	**Total carbohydrate**	**Dietary fiber**	**Energy (kcal)**
BGC	1.62 ± 0.13^c^	3.58 ± 0.08^c^	18.42 ± 1.18^b^	10.39 ± 0.83^c^	65.99 ± 0.09^b^	5.74 ± 0.48^b^	448.35 ± 0.28^b^
BEC	1.92 ± 0.15^a^	4.07 ± 0.10^b^	21.11 ± 1.35^a^	14.06 ± 1.12^a^	58.84 ± 0.07^d^	6.78 ± 0.57^a^	454.46 ± 0.35^a^
BCC	1.77 ± 0.14^b^	5.89 ± 0.28^a^	21.05 ± 1.35^a^	11.86 ± 0.95^b^	59.43 ± 0.29^c^	5.29 ± 0.44^c^	453.44 ± 1.16^a^
BGF	1.19 ± 0.09^d^	3.86 ± 0.10^bc^	16.95 ± 1.08^c^	6.69 ± 0.53^d^	71.31 ± 0.16^a^	4.18 ± 0.35^d^	447.83 ± 0.30^b^

*Note:* Data are the mean ± standard deviation. Different letters in the same column are significantly different (*p* < 0.05).

Abbreviations: BCC, biscuit recipe for children′s consumption; BEC, biscuit recipe for elderly consumption; BGC, biscuit recipe for general consumption; BGF, biscuit for gluten‐free formulation.

Moisture content is a critical parameter in baked products such as biscuits, as it directly influences shelf life, texture, and microbial stability [42]. In this study, the moisture content of the biscuit samples ranged from 3.58% to 5.89%, with BCC showing the highest value and BGC the lowest. The highest value in BCC was associated with the use of date syrup, which promotes water retention and a softer texture. In contrast, lower moisture in BGC and BGF reflected the drying effect of WWF and gluten‐free flour, enhancing shelf stability [50].

Regarding fat content, BEC had the highest level (21.11/100 g), whereas BGF had the lowest (16.95/100 g) (Table 3). The increased fat content in BEC likely stems from the presence of unsaturated fatty acids in PSF [51]. In contrast, the lower fat level in BGF may be explained by the use of gluten‐free flours such as RF, OF, and PSF, which contribute less fat compared to WWF [43, 52, 53]. MP is also valued for its low fat and high content of protein, carbohydrate, fiber, organic acids, vitamins, and minerals. Izzreen et al. [28] found that adding MP to bread increased protein, fiber, and antioxidant activity.

Protein content also differed statistically significantly across formulations (*p* < 0.05). According to Table 3, the highest protein level was detected in BEC (14.06/100 g), while the lowest was in BGF (6.69/100 g), which may be due to the absence of WG and WWF, both of which are key protein sources in traditional formulations. This reduction is also related to the elimination of gluten, a major storage protein complex in wheat that contributes significantly to the total protein content. Moreover, the substitution of wheat‐based ingredients with RF and OF in the gluten‐free formulation resulted in a lower overall protein value, as these gluten‐free flours generally contain less protein and have different amino acid compositions. The elevated protein content in BEC suggests that PSF enriched the protein profile of the formulation [48, 54]. Pumpkin seed (*Cucurbita pepo*), an edible seed and by‐product of pumpkin fruit, is a rich source of protein (39.35/100 g). A study reported that gluten‐free biscuits made with 30% PSF contained 17.50/100 g protein, highlighting the ingredient′s potential in bakery applications [55].

Total carbohydrate content also differed statistically significantly among samples (*p* < 0.05). The highest carbohydrate level was found in BGF (71.31/100 g), followed by BGC (65.99/100 g) and BCC (59.43/100 g), while BEC had the lowest (58.84/100 g) (Table 3). This reduction in carbohydrate content in BEC may be linked to higher fat and protein contributions from nutrient‐dense ingredients like PSF [51]. Conversely, the elevated carbohydrate content in BGF can be associated with gluten‐free flours like rice and oat, which are primarily starch‐based. In gluten‐free product development, potato, corn, and rice starches, along with RF, corn flour, buckwheat flour, and OF, are frequently used. Although OF and bran can enhance the nutritional value and fiber content of gluten‐free biscuits, high substitution levels may negatively affect consumer acceptance. Oat and rice are typically higher in starch compared to whole wheat [56]. A similar study suggested that incorporating stevia and flaxseed into biscuit formulations enhances their nutritional profile and sensory acceptance [57]. Stevia extract can replace sucrose to reduce sugar content without compromising taste and may support obesity management [24].

Dietary fiber content also showed significant variation. BEC exhibited the highest fiber content (6.78/100 g), followed by BGC (5.74/100 g) and BCC (5.29/100 g), with the lowest fiber content in BGF (4.18/100 g) (Table 3). The high fiber level in BEC is likely due to the inclusion of PSF. In contrast, the lower fiber content in BGF can be attributed to the dominant use of RF and OF, which typically contribute less dietary fiber than whole wheat [42].

Energy content of the biscuit samples ranged from 447.83 to 454.46 kcal/100 g, with BEC exhibiting the highest value. This can be attributed to its elevated fat and protein content, both of which contribute significantly to total caloric value. The inclusion of PSF in BEC likely enhanced lipid content, thereby increasing energy density [58]. In contrast, BGF had the lowest energy value, potentially due to its lower fat and protein levels associated with gluten‐free flours. These variations suggest that flour type and fat–protein ratios are key drivers of energy content in functional biscuit formulations [59].

Additionally, combining PSF with wheat flour was found to increase protein, fiber, zinc, iron, and antioxidant activity while maintaining high acceptability [54]. A study using mulberry juice and syrup instead of sucrose in biscuits demonstrated improved mineral and antioxidant content [60].

Similarly, incorporating Ajwa date paste at 0%, 10%, 20%, and 40% substitution levels in biscuits increased protein and iron while reducing fat, carbohydrate, and energy content [61].

### 3.3. The Changes in Total Phenolic Content and DPPH Scavenging Activity During In Vitro Gastrointestinal Digestion

Cereals serve as a primary source of starch, dietary fiber, amino acids, vitamins, and minerals necessary for human health. In addition to these macronutrients and micronutrients, cereals also contain bioactive components, including phenolic compounds, which are known for their antioxidant properties and potential health benefits [62]. In specific formulations, different kinds of food ingredients are incorporated into cereal‐based bakery products to increase the phenolic content [63, 64]. Nevertheless, polyphenolic compounds must first be effectively released from the food matrix during the digestive process to exert beneficial health effects. Furthermore, these bioactive components must demonstrate high bioaccessibility to ensure their absorption and subsequent bioactivity in the human body [65]. In this study, the bioaccessible fraction of polyphenols from different biscuit samples was evaluated after in vitro gastrointestinal digestion. Table 4 summarizes the total phenolic content of biscuits before and after digestion.

**Table 4:** In vitro gastrointestinal digestion of total phenolic content of biscuit samples in simulated gastric fluid (SGF) and simulated intestinal fluid (SIF).

	**Total phenolic content (mg GAE/kg sample)**		
	**Before digestion**	**SGF (after gastric digestion)**	**SIF (after intestinal digestion)**
BGC	15.79 ± 0.38^c,A^	9.57 ± 0.76^c,B^	16.53 ± 3.29^c,A^
BEC	19.55 ± 0.26^c,B^	12.75 ± 1.09^b,C^	41.78 ± 0.69^b,A^
BCC	32.45 ± 2.48^a,B^	19.36 ± 0.28^a,C^	50.77 ± 4.40^a,A^
BGF	26.74 ± 1.74^b,B^	19.62 ± 1.53^a,C^	41.60 ± 2.93^b,A^

*Note:* Data are the mean ± standard deviation. Different lowercase letters (a–c) for the columns based on the Tukey test indicate a significant difference (*p* < 0.05) between the total phenolic content of biscuits, while different uppercase letters (A–C) for the rows based on the Tukey test indicate a significant difference (*p* < 0.05) between the total phenolic content of biscuits at different digestion phases.

Abbreviations: BCC, biscuit recipe for children′s consumption; BEC, biscuit recipe for elderly consumption; BGC, biscuit recipe for general consumption; BGF, biscuit for gluten‐free formulation.

Among the undigested biscuit samples, the highest total phenolic content was observed in the BCC sample. This can be attributed to the inclusion of date syrup as a sweetener in the formulation of the BCC sample, because dates, both as a fruit and as a syrup, have been found to be a good source of phenolic compounds [66]. After gastric digestion, the total phenolic content of BGC, BEC, BCC, and BGF samples decreased by 39.38%, 34.78%, 40.34%, and 26.64%, respectively. It is thought that the high acidity of the stomach environment plays an important role in the decrease of the phenolic content [67]. According to Pinto et al. [65], cookies enriched with chestnut shell extract showed a 44% reduction in phenolic content after gastric digestion.

After intestinal digestion, the total phenolic content in all samples except BGC increased compared to undigested biscuits. During intestinal digestion, pancreatin–bile salts facilitate the breakdown of nutrients. This process can lead to the release of bound phenolics from food matrices under longer digestion times. Additionally, at neutral pH levels typically found in the intestines, certain phenolic compounds may become more soluble compared to their behavior in acidic conditions present in the stomach [68]. In addition to this, the aromatic rings of phenolic compounds may undergo structural modifications as the stomach and intestine transition move from an acidic to an alkaline environment, which increases the phenolic content [69]. Similar findings were reported by Gulsunoglu‐Konuskan et al. [70]. The researchers indicated that after intestinal digestion, the phenolic content in gluten‐free muffin samples increased by 1.4 to twofold compared to undigested samples. In our study, the total phenolic content of the BGF sample produced as a gluten‐free biscuit increased by 1.55‐fold after intestinal digestion. It is thought that the OF and MF used in the biscuit formulation are the source of the phenolic compounds found in the BGF samples [71, 72].

The DPPH scavenging activity of the biscuit samples before and after digestion is shown in Table 5. It was found that the samples with the highest DPPH activity before digestion were the BCC and BGF samples. The higher phenolic content of these two biscuits compared to other samples resulted in higher antioxidant activity. Phenolic compounds are known to have high antioxidant capacity [73]. The formulation of the BGF sample includes ingredients such as MF, OF, and RF, all of which are recognized for their potential to enhance antioxidant activity [28, 74, 75]. After gastric digestion, DPPH activity decreased significantly in all samples, but the highest value was observed in the BGF sample. RF in BGF formulation contains large amounts of amylose. Amylose has a firm molecular structure due to the tight packing of its functional groups, which may make it resistant to digestive enzymes [76]. After intestinal digestion, a significant increase in DPPH activities was observed in BEC and BGC samples compared to their nondigestible forms (*p* < 0.05). Pinto et al. [65] observed a 2.4‐fold increase in DPPH activity after intestinal digestion of cookies enriched with chestnut shell extract. In our study, the highest increase in antioxidant activity after intestinal digestion was observed in BEC samples, by 1.4‐fold.

**Table 5:** In vitro gastrointestinal digestion of DPPH scavenging activity of biscuit samples in simulated gastric fluid (SGF) and simulated intestinal fluid (SIF).

	**DPPH scavenging activity (mg Trolox/kg sample)**		
	**Before digestion**	**SGF (after gastric digestion)**	**SIF (after intestinal digestion)**
BGC	69.10 ± 1.92^b,A^	40.16 ± 1.12^c,B^	74.17 ± 2.79^c,A^
BEC	76.58 ± 2.88^b,B^	46.22 ± 1.61^b,C^	103.40 ± 0.31^b,A^
BCC	106.69 ± 1.92^a,B^	42.91 ± 0.26^bc,C^	112.48 ± 0.33^ab,A^
BGF	105.55 ± 4.16^a,A^	69.19 ± 0.73^a,B^	118.28 ± 4.96^a,A^

*Note:* Data are the mean ± standard deviation. Different lowercase letters (a–c) for the columns based on the Tukey test indicate a significant difference (*p* < 0.05) between the DPPH scavenging activity of biscuits, while different uppercase letters (A–C) for the rows based on the Tukey test indicate a significant difference (*p* < 0.05) between the DPPH scavenging activity of biscuits at different digestion phases.

Abbreviations: BCC, biscuit recipe for children′s consumption; BEC, biscuit recipe for elderly consumption; BGC, biscuit recipe for general consumption; BGF, biscuit for gluten‐free formulation.

The absence of in vivo digestibility experiments to verify in vitro results is a significant limitation. However, it is thought that the in vitro results from our investigation have the potential to guide further in vivo digestibility studies.

### 3.4. Microbiological Properties of Samples

High levels of bacteria and molds/yeasts can cause food spoilage and foodborne illness [77]. Therefore, the enumeration of these microorganisms in food is important for ensuring food safety and quality.

On Day 0, the total number of mesophilic aerobic bacteria and mold/yeast detected was similar in the BGC group, with numbers of 0.23 ± 0.09 and 0.26 ± 0.02 log CFU/g, respectively. However, no bacteria or mold/yeast were present in the BEC, BCC, or BGF groups on Day 0 (Table 6).

**Table 6:** Microbiological shelf life analysis.

	**Total plate count (log CFU/g)**			**Mold and yeast count (log CFU/g)**		
	**Day 0**	**Day 15**	**Day 30**	**Day 0**	**Day 15**	**Day 30**
BGC	0.23 ± 0.09^aA^	n.d.	0.26 ± 0.02^bA^	0.26 ± 0.02^aB^	0.20 ± 0.03^bB^	0.49 ± 0.16^bA^
BEC	n.d.	0.99 ± 0.13^aA^	0.10 ± 0.02^bB^	n.d.	0.23 ± 0.01^bB^	0.53 ± 0.04^bA^
BCC	n.d.	0.10 ± 0.01^bA^	n.d.	n.d.	0.10 ± 0.02^bB^	0.30 ± 0.03^bA^
BGF	n.d.	0.88 ± 0.09^aA^	0.63 ± 0.15^aA^	n.d.	0.57 ± 0.09^aB^	1.71 ± 0.13^aA^

*Note:* Data are the mean ± standard deviation. Different lowercase letters (a–c) in the same column indicate the difference between samples. Different capital letters (A–C) on the same row indicate the difference between days (*p* < 0.05).

Abbreviations: BCC, biscuit recipe for children′s consumption; BEC, biscuit recipe for elderly consumption; BGC, biscuit recipe for general consumption; BGF, biscuit for gluten‐free formulation; n.d., not determined.

On Day 15, the number of bacteria in the BEC and BGF groups (0.99 ± 0.13 and 0.88 ± 0.09 log CFU/g, respectively) was found to be higher than in the BCC group (0.10 ± 0.01 log CFU/g). At this time point, no bacteria were found in the BGC group. On Day 30, the bacterial counts in the groups of BGC, BEC, and BGF were 0.26 ± 0.02, 0.10 ± 0.02, and 0.63 ± 0.15 log CFU/g, respectively. No bacteria were detected in the BCC group on Day 30 (Table 6).

On the 15th day, it was determined that there was little difference in the number of mold/yeast compared to Day 0 in the BGC group. However, the number of mold/yeast in the BEC (0.23 ± 0.01 log CFU/g) and BGF group samples was slightly higher (0.57 ± 0.09 log CFU/g) on Day 15 than in the BCC group (0.10 ± 0.02 log CFU/g). The mold/yeast counts on Day 30 were recorded as 0.49 ± 0.16, 0.53 ± 0.04, 0.30 ± 0.03, and 1.71 ± 0.13 log CFU/g for the BGC, BEC, BCC, and BGF groups, respectively (Table 6). The highest mold/yeast count was observed in the BGF group among all tested groups. The number of molds/yeasts detected in the BGF sample on 30 days remains below legal limits but has increased significantly compared to previous test time points. This suggests that the microbial stability of the product is decreasing over time by means of molds/yeasts, which may result in potential quality loss or an increased risk of mycotoxins, especially if the shelf life is extended.

Aderinola et al. [18] reported that total viable counts of biscuit samples, which were supplemented with *Moringa oleifera* seeds (2%, 4%, and 6%), were 2, 7, and 7 × 10^3^ CFU/g, respectively. The researchers did not observe any yeast/mold in all the biscuit samples, and they indicated that the levels of microbiological parameters were within the safe range for baked products. In our samples, bacterial counts were found to be very low. This may be attributed to the content of WG. Mahmoud et al. [78] reported the antibacterial activity of WG extract at different concentrations against *Listeria monocytogenes*, *Staphylococcus aureus*, *Salmonella enterica*, and *Escherichia coli*.

One of the reasons for the higher number of molds/yeast in the BGF group may be the presence of a mixture of MF, RF, and OF. As MF contains high levels of fructose, the number of molds/yeasts may be higher. Ji et al. [79] investigated the yeast/mold count of cakes made from RF and reported that the yeast/mold count increased slowly throughout the storage period but remained below < 100. On the other hand, the date juice used in the recipes of the BCC group actually contained sugar, so we expected the effect to be similar to that of the BGF group, but date fruit has been previously reported to have various biological activities, one of which is antifungal activity [80]. Therefore, the difference between these two groups, BCC and BGF, may be due to this reason. The International Commission on Microbiological Specifications for Microorganisms in Foods (ICMSF) has reported that a total plate count lower than 4 log CFU/g is considered to be satisfactory in terms of microbiological quality [81]. Conversely, a total plate count that exceeds 6 log CFU/g is considered to be unacceptable. In view of the findings of this study, it can be concluded that the number of microorganisms was found to be well below the specified limit. This finding indicates that the samples were microbiologically safe for human consumption. When a comparison is made between groups based on the microbiological shelf‐life analysis, BCC demonstrated the optimal microbial stability and consequently the longest shelf life, whereas BGF exhibited elevated microbial counts, indicating a reduced shelf life in comparison to the other formulations. However, it should be noted that even the numbers in the BGF group are well below these specified limits.

### 3.5. Sensory Properties of Biscuit Samples

The sensory evaluation results of the biscuit samples are shown in Table 7. In terms of color, the BGF sample received the highest rating (7.88), while the BCC received the lowest (5.60). BGF, which contains MP, showed high *a* ^∗^ and *b* ^∗^ values and low *L* ^∗^ values, indicating a more reddish‐yellow hue and a darker color [27]. In contrast, the addition of iron in BCC likely contributed to its darker color, as the lower *b* ^∗^ value suggests a shift toward more green tones [82]. This was also reflected in the sensory evaluation, where the color parameter received the lowest score. Similarly, the lowest odor score (5.72) was recorded for BCC, whereas BGC received the highest (7.84) due to the iron fortification.

**Table 7:** Sensory analysis results.

	**BCG**	**BEC**	**BCC**	**BGF**
Color	7.60 ± 0.96^ab^	6.88 ± 0.97^b^	5.60 ± 1.71^c^	7.88 ± 1.36^a^
Odor	7.84 ± 0.8^0a^	6.60 ± 1.35^bc^	5.72 ± 1.51^c^	7.48 ± 1.50^ab^
Taste	8.00 ± 1.08^a^	6.76 ± 1.33^b^	5.04 ± 1.46^c^	7.44 ± 1.89^ab^
Crispness	8.04 ± 0.89^a^	6.68 ± 1.18^b^	5.28 ± 1.65^c^	8.20 ± 1.29^a^
Softness	7.72 ± 1.17^a^	7.32 ± 1.31^ab^	6.52 ± 1.45^c^	8.16 ± 1.03^a^
Stickiness	7.64 ± 0.95^a^	7.08 ± 1.15^a^	5.92 ± 1.35^b^	7.80 ± 1.35^a^
Overall acceptability	7.92 ± 0.91^a^	6.92 ± 1.04^b^	5.64 ± 1.22^c^	7.76 ± 1.67^ab^

*Note:* Data are the mean ± standard deviation. Different letters in the same line are significantly different (*p* < 0.05).

Abbreviations: BCC, biscuit recipe for children′s consumption; BEC, biscuit recipe for elderly consumption; BGC, biscuit recipe for general consumption; BGF, biscuit for gluten‐free formulation.

In flavor evaluation, BGC received the highest score (8.00), while BCC had the lowest (5.04), likely due to the metallic taste associated with iron fortification. A study investigated the sensory acceptability of iron and zinc fortification through sensory evaluations conducted with 35 women. The results revealed that participants were more willing to consume samples prepared with the lowest iron concentration (1 g/L), while higher iron levels negatively affected sensory properties [83].

Crispness scores ranged from 5.28 (BCC) to 8.20 (BGF). There was no statistically significant difference between BGF and BGC (*p* > 0.05), which is a promising finding for BGF. Similarly, softness scores showed no statistical difference between BGC (7.72) and BGF (8.16), with both samples receiving the highest scores for this attribute. For stickiness, the BCC sample received the lowest score (5.92), while no significant difference was observed among the other samples (*p* > 0.05). Overall acceptability scores ranged between 5.64 and 7.92, with BCC receiving the lowest rating. These results suggest that although iron fortification can enhance nutritional value, its concentration must be carefully optimized to maintain consumer acceptance [82]. All samples received scores of 5 or higher across all parameters, suggesting they were well received and are considered acceptable for consumption. The slightly lower sensory scores observed for the BEC and BCC formulations were primarily attributed to the nutritional modifications designed for these target groups, including reduced sugar content in BEC and iron fortification in BCC. Iron fortification, while nutritionally beneficial, may impart a mild metallic or bitter taste and affect color and aroma perception, as reported in previous studies [84, 85]. Despite these differences, all biscuit formulations remained within the acceptable sensory quality range, confirming their suitability for the intended population groups.

In summary, the BEC formulation presented the highest levels of ash, fat, protein, and dietary fiber, indicating a nutritionally enriched profile. Meanwhile, the BGF sample, although offering a gluten‐free alternative, exhibited the highest carbohydrate content but the lowest levels of key nutrients, revealing certain nutritional limitations compared to wheat‐based formulations. These findings underscore the crucial role of ingredient selection in shaping the nutritional profile of biscuits and highlight the importance of tailored functional product development for specific dietary needs. However, it should be noted that the sensory panel in this study consisted of students and academicians from the Department of Gastronomy and Culinary Arts. While this group provided consistent and informed evaluations, it may not fully represent the broader consumer population. Future studies should therefore involve participants from diverse demographic backgrounds to better capture real‐world consumer perceptions and preferences.

### 3.6. Radar Diagram Interpretation

**Figure 2:**
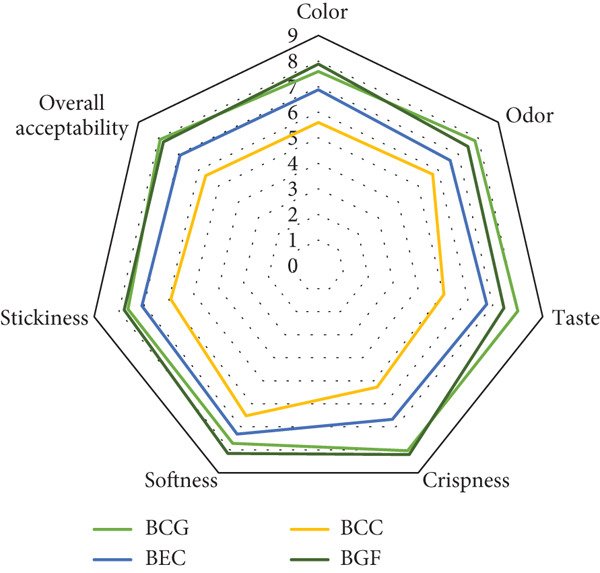
Radar diagram of biscuits based on sensory evaluation. BGC, biscuit recipe for general consumption; BEC, biscuit recipe for elderly consumption; BCC, biscuit recipe for children′s consumption; BGF, biscuit for gluten‐free formulation.

The radar diagram (Figure 2) offers a visual summary of how the biscuit formulations performed across key sensory attributes. Among the samples, BGF and BGC displayed broader radar profiles, reflecting higher overall acceptability and well‐balanced color, flavor, and texture scores. Conversely, BCC presented a noticeably smaller area, mainly due to its lower ratings in color and flavor, which align with the metallic aftertaste and darker tone resulting from iron fortification. The nearly symmetrical shapes of BGF and BGC indicate a more uniform sensory quality, whereas the uneven outline of BCC highlights its weaker points. These visual trends are in full agreement with the numerical results given in Table 7, confirming the consistency between graphical and statistical evaluations.

### 3.7. Correlation and Cluster Analysis

**Figure 3:**
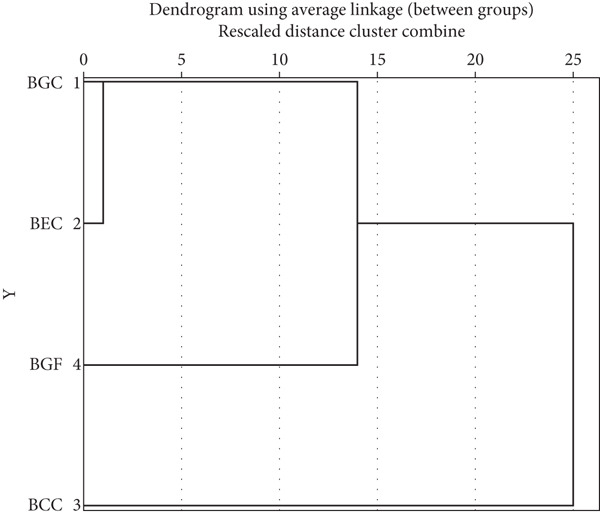
Dendrogram about hierarchical cluster analyses. BGC, biscuit recipe for general consumption; BEC, biscuit recipe for elderly consumption; BCC, biscuit recipe for children′s consumption; BGF, biscuit for gluten‐free formulation.

Correlation analysis showed that certain physicochemical and sensory parameters were closely related (Table 8). For instance, hardness was positively associated with both taste and general acceptability, suggesting that firmer texture contributed positively to sensory quality. Similarly, diameter and color attributes (*L* ^∗^, *a* ^∗^, and *b* ^∗^) showed strong correlations with crispiness and odor, underlining the role of appearance and structure in consumer perception. On the other hand, both energy content and total phenolic content exhibited negative correlations with acceptability scores (*R* ^2^ = −0.79 and −0.71, respectively). This inverse relationship can be explained by the intrinsic sensory properties of phenolic compounds, which are often associated with bitterness, astringency, and darker coloration. Polyphenol–protein interactions can also modify texture and mouthfeel, while oxidation reactions involving phenolics may suppress desirable volatile aroma compounds. These mechanisms collectively reduce the perceived pleasantness and overall acceptability of enriched formulations, despite their nutritional and functional benefits [86]. HCA was performed using the average linkage (between‐groups) method and Euclidean distance as the similarity measure, since this combination provides balanced cluster formation and minimizes the effect of outliers. The dendrogram (Figure 3) was labeled with sample names (BGC, BEC, BCC, and BGF) to enhance interpretability. The analysis grouped the samples into two main clusters: BGC, BEC, and BCC showed close similarity, while BGF appeared as a separate cluster. This separation is likely due to its gluten‐free formulation, which differs in both ingredient composition and textural behavior [87]. These results confirm that multivariate analyses such as correlation and HCA can effectively reveal similarities and differences among biscuit formulations (Table 8, Figure 3).

**Table 8:** Pearson′s correlation coefficients among the physicochemical, textural, sensory, and bioactive properties of the biscuit samples (variables were coded from 1 to 24 for clarity).

	**1**	**2**	**3**	**4**	**5**	**6**	**7**	**8**	**9**	**10**	**11**	**12**	**13**	**14**	**15**	**16**	**17**	**18**	**19**	**20**	**21**	**22**	**23**	**24**	**25**
1. Hardness	1.000	−0.695	0.721	−0.658	0.805	−0.095	0.771	−0.227	0.603	0.761	0.897	0.915	0.797	0.657	0.783	0.863	−0.860	−0.182	−0.189	−0.511	0.162	0.438	−0.617	−0.901	−0.788
2. Fracturability		1.000	−**0.983** ^∗^	0.560	−0.867	−0.040	−0.335	−0.322	−0.939	−**0.968** ^∗^	−0.871	−0.919	−0.930	−**0.958** ^∗^	−**0.976** ^∗^	−0.938	**0.960** ^∗^	0.524	0.452	0.705	0.106	−0.679	0.612	0.647	0.399
3. Diameter			1.000	−0.438	0.793	−0.145	0.267	0.410	**0.979** ^∗^	**0.997** ^∗∗^	0.926	0.939	**0.979** ^∗^	**0.992** ^∗∗^	**0.996** ^∗∗^	**0.970** ^∗^	−0.942	−0.646	−0.591	−0.820	−0.254	0.795	−0.748	−0.592	−0.326
4. Thickness				1.000	−0.895	−0.686	−0.891	0.579	−0.250	−0.427	−0.434	−0.591	−0.376	−0.320	−0.481	−0.498	0.699	−0.403	−0.459	−0.118	−0.757	0.179	−0.108	0.917	0.918
5. Spread ratio					1.000	0.392	0.737	−0.189	0.655	0.784	0.754	0.867	0.741	0.709	0.820	0.819	−0.942	−0.048	0.017	−0.328	0.387	0.274	−0.299	−0.916	−0.784
6. Baking loss						1.000	0.448	−0.578	−0.283	−0.196	−0.308	−0.119	−0.296	−0.241	−0.145	−0.195	−0.074	0.736	0.813	0.674	0.863	−0.686	0.754	−0.345	−0.377
7. *L* ^∗^							1.000	−0.767	0.069	0.292	0.437	0.543	0.295	0.148	0.343	0.428	−0.570	0.479	0.479	0.147	0.747	−0.227	0.021	−0.934	−**0.997** ^∗∗^
8. *a* ^∗^								1.000	0.585	0.389	0.223	0.120	0.383	0.519	0.338	0.250	−0.082	−0.904	−0.875	−0.711	−0.906	0.768	−0.558	0.492	0.729
9. *b* ^∗^									1.000	**0.974** ^∗^	0.883	0.867	**0.961** ^∗^	**0.997** ^∗∗^	**0.961** ^∗^	0.923	−0.854	−0.784	−0.732	−0.899	−0.440	0.888	−0.807	−0.418	−0.129
10. Color										1.000	**0.953** ^∗^	**0.957** ^∗^	**0.992** ^∗∗^	**0.988** ^∗^	**0.998** ^∗∗^	**0.985** ^∗^	−0.944	−0.653	−0.607	−0.840	−0.267	0.811	−0.787	−0.609	−0.347
11. Odor											1.000	**0.981** ^∗^	**0.979** ^∗^	0.909	**0.954** ^∗^	**0.987** ^∗^	−0.921	−0.580	−0.566	−0.823	−0.220	0.776	−0.848	−0.697	−0.474
12. Flavor/taste												1.000	**0.962** ^∗^	0.902	**0.967** ^∗^	**0.991** ^∗∗^	−**0.977** ^∗^	−0.459	−0.429	−0.724	−0.057	0.673	−0.733	−0.796	−0.586
13. Crispiness													1.000	**0.975** ^∗^	**0.987** ^∗^	**0.990** ^∗^	−0.924	−0.680	−0.648	−0.876	−0.314	0.843	−0.850	−0.602	−0.344
14. Softness														1.000	**0.980** ^∗^	0.948	−0.892	−0.738	−0.686	−0.878	−0.375	0.861	−0.796	−0.488	−0.207
15. Stickiness															1.000	**0.988** ^∗^	−**0.962** ^∗^	−0.606	−0.558	−0.807	−0.208	0.774	−0.757	−0.653	−0.398
16. General acceptability																1.000	−**0.963** ^∗^	−0.568	−0.535	−0.801	−0.178	0.759	−0.790	−0.710	−0.475
17. Moisture																	1.000	0.367	0.315	0.625	−0.064	−0.578	0.597	0.825	0.621
18. Ash																		1.000	**0.992** ^∗∗^	0.938	0.902	−**0.964** ^∗^	0.859	−0.172	−0.442
19. Protein																			1.000	0.934	0.926	−**0.956** ^∗^	0.882	−0.196	−0.451
20. Fat																				1.000	0.731	−**0.996** ^∗∗^	**0.967** ^∗^	0.167	−0.109
21. Dietary fiber																					1.000	−0.775	0.677	−0.544	−0.735
22. Total carbohydrate																						1.000	−0.948	−0.092	0.188
23. Energy																							1.000	0.240	0.002
24. Total phenolic content																								1.000	**0.955** ^∗^
25. DPPH scavenging activity																									1.000

*Note:* Values printed in bold correspond to statistically significant correlations.

^*^Correlation is significant at the 0.05 level (2‐tailed). ^**^Correlation is significant at the 0.01 level (2‐tailed).

## 4. Conclusion

This study is aimed at exploring the development of biscuit formulations that may serve as nutritionally supportive options for various population groups, particularly under emergency and disaster conditions. Four formulations were prepared to address the needs of the general population (BGC), elderly individuals (BEC), children (BCC), and individuals following a gluten‐free formulation (BGF). Among these, the BEC formulation demonstrated a notably enhanced nutritional profile, especially in terms of protein, fat, dietary fiber, and ash content, largely due to the use of PSF. In contrast, although the BGF formulation offered a gluten‐free alternative, it showed relatively lower levels of key nutrients and signs of reduced microbial stability over time.

The results also indicated that gluten‐free biscuits, when carefully formulated, can achieve favorable sensory and physical properties. The BGF sample, in particular, was well received in terms of color and softness, highlighting its potential for consumer acceptance. In vitro digestion studies showed that samples enriched with bioactive ingredients, such as MP and date syrup, maintained or improved their phenolic content and antioxidant activity, suggesting added functional benefits.

Despite these promising findings, further research is recommended to optimize the nutritional quality and microbial safety of gluten‐free products intended for long‐term storage. Future work should examine alternative preservation techniques, assess the sensory effects of different fortification approaches, and conduct shelf‐life studies under conditions relevant to emergency use. Expanding bioavailability assessments and consumer testing across target groups would also contribute to the effective design of functional food products tailored for vulnerable populations.

The formulated biscuits also hold promise as practical, shelf‐stable, and nutrient‐dense food options that could support nutritional needs in resource‐limited, emergency, or postdisaster contexts. Their balanced macronutrient composition and inclusion of locally available ingredients suggest potential application in food aid or community nutrition programs.

Nevertheless, this study is limited by its laboratory‐scale production, relatively small sensory panel, and the absence of long‐term storage stability testing. Future research should therefore focus on validating the formulations under real‐world conditions and evaluating their sensory and nutritional performance during extended storage.

**Nomenclature**
BGC: biscuit recipe for general consumption
BEC: biscuit recipe for elderly consumption
BCC: biscuit recipe for children′s consumption
BGF: biscuit for gluten‐free formulation

## Ethics Statement

The sensory evaluation of biscuit samples in the present study was conducted with the approval of Maltepe University′s Ethics Committee (Decision No: 2024/23‐16), granted on 24.12.2024. All participants provided written informed consent prior to participation. The study complied with all relevant ethical standards involving human participants. All authors were compliant and followed the ethical guidelines, according to the requirements of journal.

## Conflicts of Interest

The authors declare no conflicts of interest.

## Author Contributions

Tuğçe Boğa: conceptualization, writing (original draft), methodology, software, formal analysis, and experimental design. Kübra Topaloğlu Günan: conceptualization, writing (original draft), methodology, and experimental design. Seda Çakmak Kavsara: conceptualization, writing (original draft), methodology, and experimental design. Didem Berber: formal analysis, writing (original draft), and supervision. Özlem Aktürk Gümüşay: formal analysis, writing (original draft), and supervision.

## Funding

This study was funded by Maltepe University, 2023HDAP03.

## Data Availability

The data is available on request from the authors.
